# Critical contribution of moisture to the air quality deterioration in a warm and humid weather

**DOI:** 10.1038/s41598-023-31316-1

**Published:** 2023-03-14

**Authors:** Woosuk Choi, Chang-Hoi Ho, Ka-Young Kim

**Affiliations:** 1 Department of Data Science, Sejong University, 209 Neungdong-ro, Gwangjin-gu Seoul, 05006 Republic of Korea; 2 School of Earth and Environmental Sciences, Seoul National University, 1 Gwanak-ro, Gwanak-gu Seoul, 08826 Republic of Korea

**Keywords:** Climate sciences, Environmental sciences, Natural hazards

## Abstract

The deterioration of air quality that threatens human health is recognized as focal compound hazard. Here, decisive thermodynamic conditions for activation of secondary aerosol formation have been investigated focused on Korea. In a dry environment with relative humidity < 60%, gas phase reaction to form fine particles depended largely on surface temperature. In a wet environment (relative humidity ≥ 60%), however, aqueous phase reaction of secondary inorganic aerosols, which are sulfate, nitrate, and ammonium, accounting for 67% of the total aerosol mass, was more activated. Thus, humidity is as important as temperature in the secondary production of aerosol; air quality often worsened when a low-pressure system was predominant over the Korean Peninsula. It is rather different from the general synoptic conditions of high concentrations of particulate matters characterized by high pressure and atmospheric stagnation. This study suggests additional favorable condition and responsible mechanism of air quality hazards that may be frequent in future.

## Introduction

The meteorological compound hazards usually refer to tropical cyclones and torrential rains on a short time scale within a couple of days, and droughts and heat waves on a relatively long time scale^1–4^. It is noted that the air quality has deteriorated utterly in many Asian countries, as the industrialization progressed over the past 20–30 years^5,6^. Severe air pollution would affect the respiratory and nervous system of people; thus, it received large social attention as much as weather disasters^5–9^. In general, air quality deterioration occurred due to coupling of natural and anthropogenic factors and could not be attributed solely to the cause of either one^10–17^.

Through the intense observation campaigns covering airborne and ground observations, and investigation of synoptic conditions for high concentrations of particulate matters (PMs) episodes, various perspectives of aerosol including direct emission, transportation, secondary generation by atmospheric chemical reactions, and changes in photochemical properties due to the aging have been examined^18–24^. Based on these understanding, local emissions from the vehicles and factories in industry sector have been regulated by the air quality management policies to reduce the average concentration of PM^25–28^. Notice that recent studies demonstrated that the importance of secondary aerosol formation by atmospheric chemical reactions was emerged, and its contribution to PMs that are ≤ 2.5 μm in diameter (PM_2.5_) concentration would be greater than 50% in some regions^29–35^. Therefore, it is necessary to understand secondary aerosol formation to reduce social damages and costs caused by air quality problem.

Chemical reactions in the atmosphere among three major secondary inorganic aerosol (SIA) species such as sulfate (SO_4_^2−^), nitrate (NO_3_^−^), and ammonium (NH_4_^+^) are known to be active in a warm and stable environment^36–40^. In other words, atmospheric conditions required for reactions are important, and the oxidation activity may depend on meteorological conditions. Previous studies evaluated the effects of weather on the air quality in the comprehensive point of view^41–43^. The present study has aimed to focus on the contribution of moisture that affects the aqueous phase reaction on the secondary aerosol formation in the Republic of Korea (hereafter Korea). The effect of humidity on the air pollution problem was quantitatively assessed and the possibility of air quality deterioration that may be caused by increased moisture in future global warming conditions was discussed.

## Methods

### Air quality and weather datasets

The hourly PM_2.5_ concentrations at 25 air quality monitoring stations in Seoul, Korea during 2007–2019 were obtained from the AirKorea website (https://www.airkorea.or.kr/) and Seoul Research Institute of Public Health and Environment^44–46^. For the quantitative diagnose of secondary aerosol formation, the three major SIA species during 2012–2018 were analyzed. These species were observed at every hour at Bulgwang station (37.61° N, 126.93° E) located inside Seoul city. Also, concentrations of gaseous aerosol precursors such as sulfur dioxide (SO_2_) and nitrogen oxide (NO_2_) were investigated. The air quality datasets during the cold season (November through next March) were used to emphasize the high PM concentration period, and their daily mean values were adopted for the consistency with atmospheric variables.

For the assessment of meteorological effects, daily mean surface air temperature, relative humidity (RH), solar radiation, and total cloud cover were obtained from the Korea Meteorological Administration (KMA). These variables were observed in Seoul automated synoptic observing system station (37.57° N, 126.97° E). To investigate large-scale atmospheric fields during the periods of high PM_2.5_ concentrations, daily mean values of geopotential height, RH, and temperature at 500-, 850-, and 1000-hPa levels from the European Centre for Medium-Range Weather Forecasts (ECMWF) Reanalysis-5 (ERA5) project data were analyzed^47,48^. The horizontal resolution of ERA5 dataset is 1.5° × 1.5° latitude and longitude. All meteorological variables were analyzed for 2007–2019.

### Quantification of gas-to-particle conversion rates of sulfur and nitrogen

To evaluate gas-to-particle conversion rates through observation data analysis, a metric that can estimate secondary aerosol formation was used. Here, gas-to-particle conversion rates were obtained by calculating the sulfur and nitrogen oxidation ratio (SOR and NOR, respectively)^49–51^. These metrics indicate the gas-to-particle conversion rates of gas phase (i.e., SO_2_ and NO_2_) to particulate phase (i.e., SO_4_^2−^ and NO_3_^−^).

$$SOR= \frac{n({SO}_{4}^{2-})}{n\left({SO}_{4}^{2-}\right)+ n({SO}_{2})}$$

$$NOR= \frac{n({NO}_{3}^{-})}{n\left({NO}_{3}^{-}\right)+ n({NO}_{2})}$$

where n(SO_4_^2−^), n(SO_2_), n(NO_3_^−^), and n(NO_2_) are the molar concentrations (μ mol m^−3^) of each chemical species. Previous studies reported that oxidations in the atmosphere are active if SOR and NOR values are higher than 0.1^52,53^.

## Results

### Active gas-to-particle conversions during high PM_2.5_ concentration episodes

The atmospheric environments during high concentration of PM_2.5_ periods in Seoul would be specified as certain active conditions for causing atmospheric chemical reactions. During whole analysis period, 210 days with a daily mean PM_2.5_ concentration of ≥ 35 μg m^−3^ were defined as high PM_2.5_ episode. The averaged PM_2.5_ concentration was 17.5 μg m^−3^ higher during these episodes than the cold season average (Table 1). As a reason of this difference, the concentrations of the three major SIA species (i.e., SO_4_^2−^, NO_3_^−^, and NH_4_^+^), which accounted for more than 50% of PM_2.5_, were much higher during the high concentration episodes. These species can actively generate PM from the gas phase through atmospheric chemical reactions, so it makes more favorable conditions of gas-to-particle conversion with greater SOR and NOR values in the high concentration episodes (0.22 for SOR and 0.11 for NOR). It is noted that the amounts of primary emission and fugitive dust would be not major contributors for causing high PM_2.5_ concentrations because they are known to not change significantly even at high concentrations episodes^32–34^. Consequently, the secondary particle formation is likely to be responsible factor for high concentration of PM_2.5_.

**Table 1:** Daily mean and standard deviation of PM_2.5_ chemical compositions, gaseous pollutants, and oxidation ratios in Seoul for the cold season (November through March) and high PM_2.5_ concentration episodes during 2012–2018.

Variables (unit)	Daily mean ± standard deviation	
	Cold season	High PM_2.5_ episode
PM_2.5_ (μg m^−3^)	28.1 ± 14.7	45.7 ± 7.9
SO_4_^2−^ (μg m^−3^)	5.0 ± 4.6	8.7 ± 4.8
NO_3_^−^ (μg m^−3^)	8.0 ± 6.5	14.1 ± 6.0
NH_4_^+^ (μg m^−3^)	4.3 ± 3.3	7.6 ± 2.9
SO_2_ (ppb)	5.8 ± 1.9	6.9 ± 2.3
NO_2_ (ppb)	33.3 ± 12.5	41.8 ± 10.8
SOR (molar ratio)	0.16 ± 0.09	0.22 ± 0.09
NOR (molar ratio)	0.07 ± 0.04	0.11 ± 0.04

### Environmental conditions for gas-to-particle conversions

**Figure 1:**
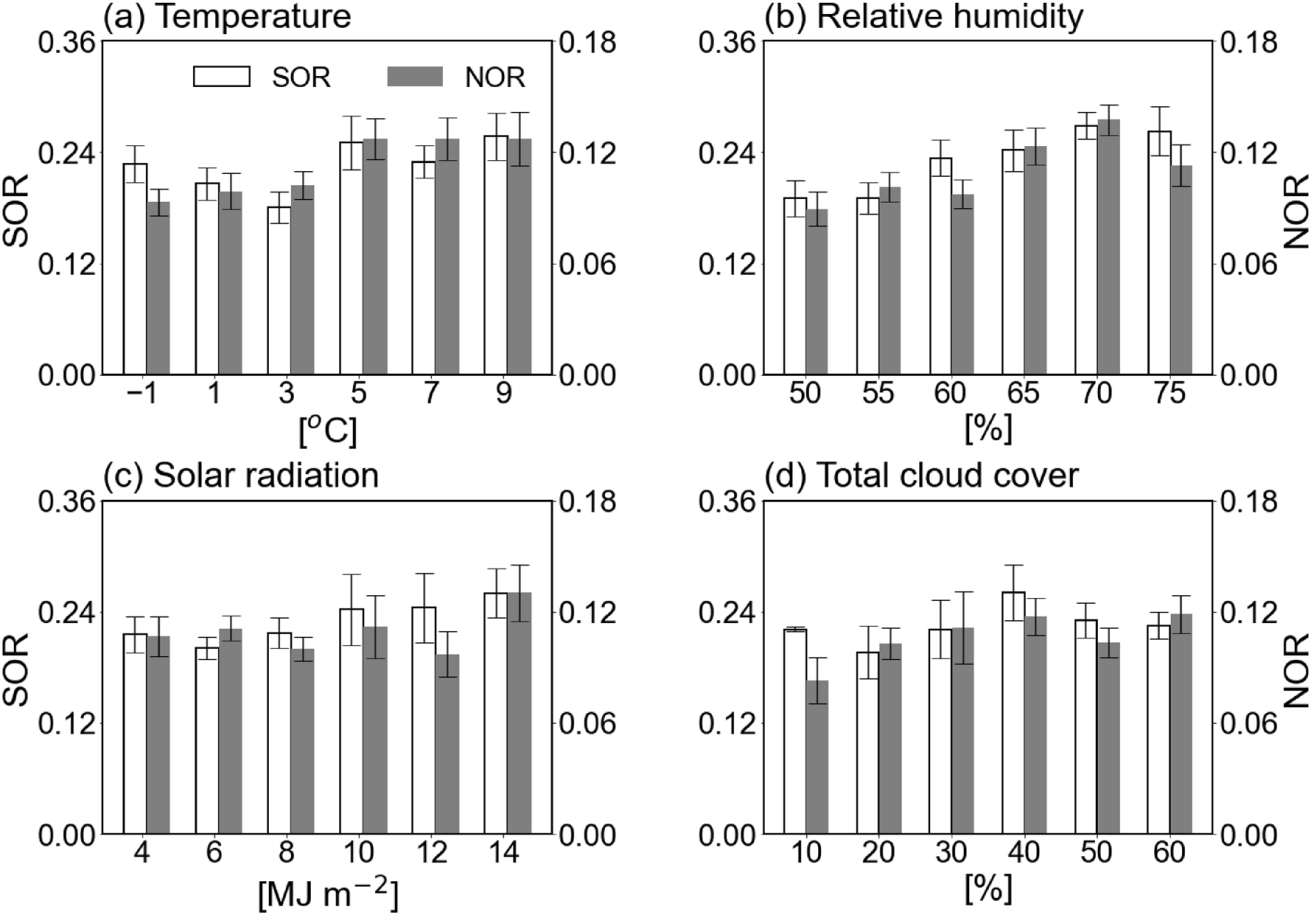
Tow oxidation ratios—SOR and NOR—corresponding to (**a**) temperature, (**b**) relative humidity (RH), (**c**) solar radiation, and (**d**) total cloud cover during high-PM_2.5_ episodes. The mean values are shown as the bars with ± 1 standard deviation.

The secondary generation of aerosol is dependent on the weather conditions, which are reaction environments, as well as the SIA concentration^15,17,22,32,39^. In general, warm condition gets most of chemical reactions active, and solar radiation promotes conversion of gaseous precursors to particle through the photochemical gas phase reaction^54,55^. On the other hand, since the amount of water vapor in the atmosphere is a major factor to determine whether to activate the aqueous phase reaction^15,31,37^, diagnosis of RH is necessary. In Fig. 1, changes in SOR and NOR according to surface temperature, RH, solar radiation, and total cloud cover were displayed. As the temperature and RH increased, the SOR and NOR values increased (Fig. 1a,b, respectively), providing favorable conditions for oxidation^49–51^. In particular, the SOR and NOR values were increased remarkably at the RH of 60%. Both oxidation ratios showed significant correlation coefficients between temperature and humidity at the 99% confidence level.

On the other hand, solar radiation, known as an essential element of photochemical reaction^15,17,52^, showed no noteworthy relationships with SOR and NOR (Fig. 1c). The correlation coefficients of SOR and NOR with solar radiation were 0.10 and 0.13, respectively, which are not statistically significant. It means that high concentration could occur sufficiently even in cloudy weather with relatively little solar radiation. In this condition, the aqueous phase reaction was more influential than the gas phase reaction as a focal mechanism for high concentration. There was no clear relationship between the total cloud cover and the two oxidation ratios (Fig. 1d). Summing up, RH would be key factor that controls oxidation along with the temperature.

**Figure 2:**
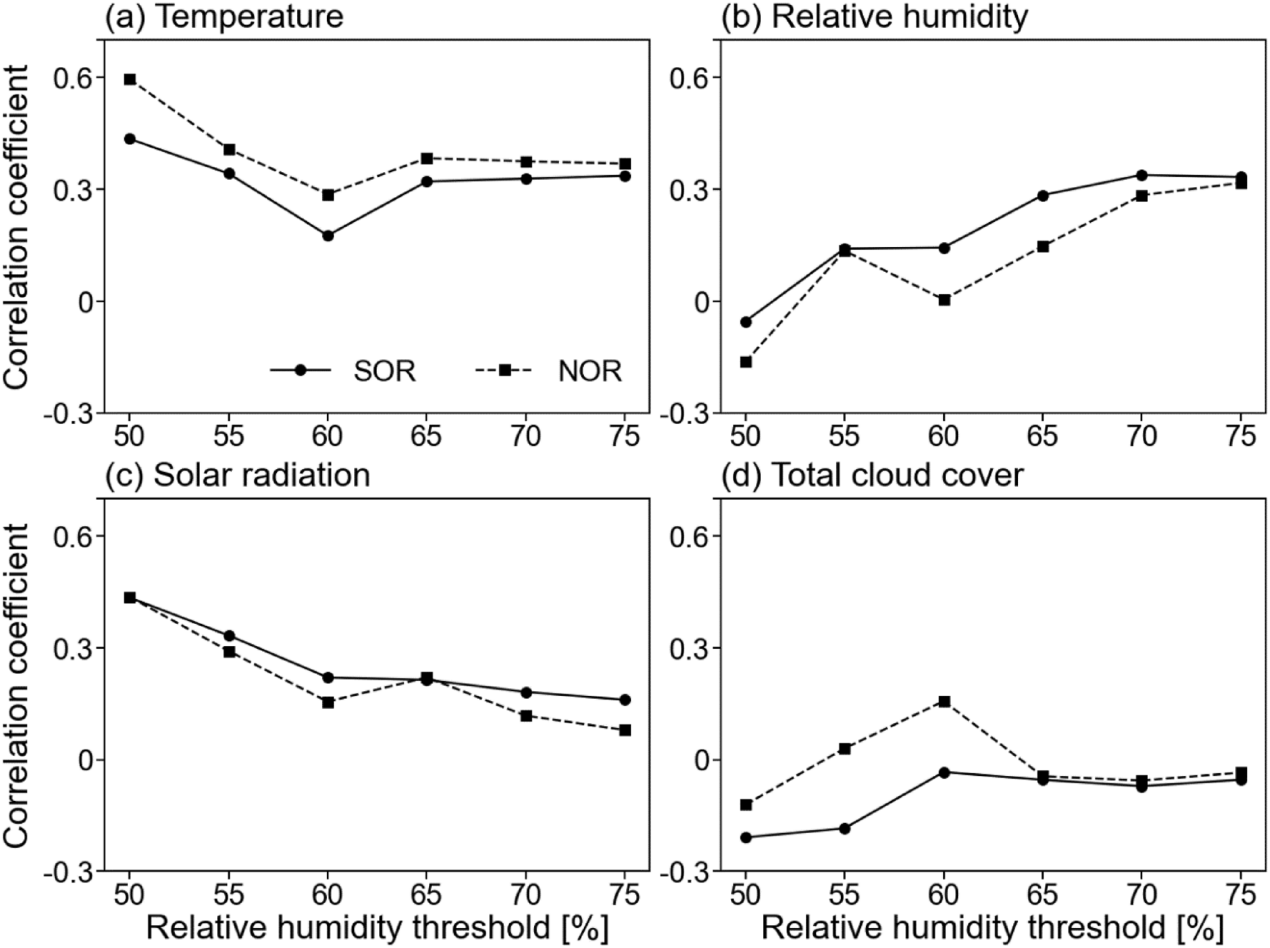
Correlation coefficients between two oxidation ratios and meteorological variables corresponding to environmental RH thresholds, (**a**) temperature, (**b**) RH, (**c**) solar radiation, and (**d**) total cloud cover.

Correlation coefficients between oxidation ratios and meteorological variables according to RH threshold were investigated to distinguish the effect of humidity on the SIA oxidation (Fig. 2). The changes in oxidization ratios were examined by changing the RH threshold from dry conditions. In a dry environment with RH < 60%, high correlation coefficients with oxidation ratios were observed in analysis of temperature and solar radiation (Fig. 2a,c, respectively). However, the higher RH (i.e., wet condition), the greater positive relationships with RH were shown (Fig. 2b). So, in the wet environment, the influence of RH was as relevant as temperature (Fig. 2a). Total cloud cover was generally less related to oxidization rates regardless of humidity thresholds (Fig. 2d). These results suggest that the production of SIA can be facilitated by aqueous phase reaction rather than photochemical reaction in humid conditions^15,31,37^. This would be a probable answer to the question of how much wet condition to convert gas to particle.

### Temporal evolutions of reaction conditions during the high PM_2.5_ concentration episodes

**Figure 3:**
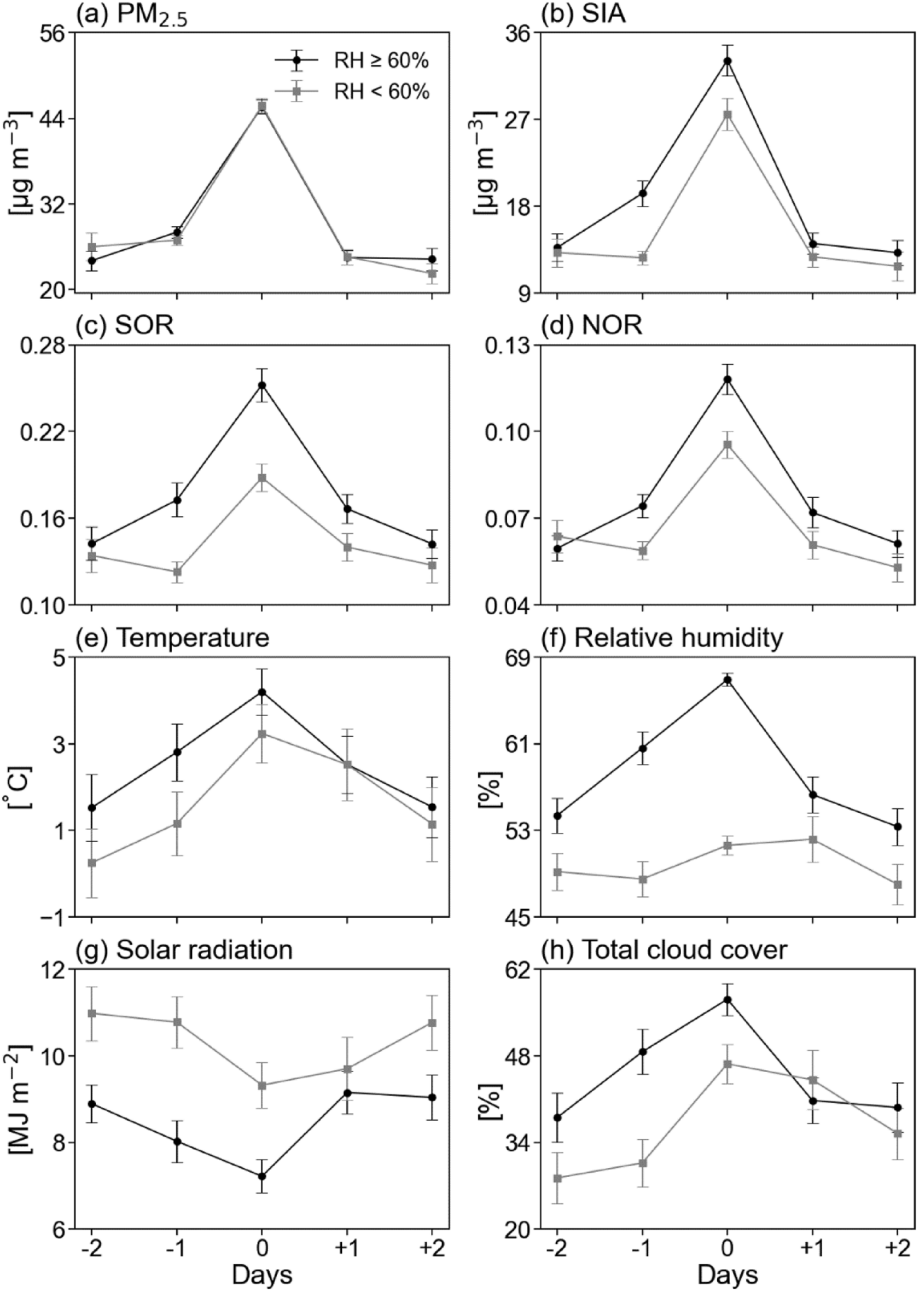
Daily mean concentrations of (**a**) PM_2.5_, (**b**) SIA, (**c**) SOR, (**d**) NOR, (**e**) temperature, (**f**) RH, (**g**) solar radiation, and (**h**) total cloud cover for the period from Day − 2 to Day + 2 of high-PM_2.5_ episodes under wet condition (RH ≥ 60%) and dry condition (RH < 60%).

Based on the day when the high PM_2.5_ concentration occurred, the changes in air pollutants including PM_2.5_, gaseous precursors, and SIAs, and meteorological variables for a total of five consecutive days were examined according to RH (Fig. 3). Here, all high PM_2.5_ episodes (i.e., whole continuous high PM_2.5_ days) are defined as Day 0. To confirm the meteorological conditions related to aqueous phase reaction, cases of high PM_2.5_ episodes were divided into two groups of humid and dry cases based on the threshold value (RH of 60%). The frequency of humid (i.e., RH ≥ 60%) and dry cases (i.e., RH < 60%) was 56% (117 episodes) and 44% (93 episodes) of the total high PM_2.5_ episodes, respectively, for the cold seasons of 2007–2019.

During the analyzed five consecutive days (i.e., Day − 2 to Day + 2), the PM_2.5_ concentration on Day 0 was approximately 20 μg m^−3^ higher than before and after regardless of humidity conditions (Fig. 3a). The SIA concentration was noticeably higher in humid condition than in dry condition on Day − 2 to Day 0 (Fig. 3b). However, gaseous precursors under the humid condition were lower than the dry condition. As a result, NOR and SOR values in the humid condition were higher than those in the dry condition, resulting in favorable environments for secondary generation (Fig. 3c,d).

To understand these changes in the concentration of air pollutants, changes in meteorological variables have been examined in the following. The high PM_2.5_ concentration episodes during the cold season occur when the temperature was relatively warm regardless of humidity condition (Fig. 3e). In the humid case, however, RH increased during the period Day − 2 to Day 0, becoming the highest on Day 0 and lower thereafter (Fig. 3f). This means that the increase in RH can contribute to the development of high concentration through aqueous phase reaction^15,17,31,37^. By contrast, dry cases did not show notable increase (or decrease) of RH, and high concentrations seem to be occurred due to other causes not the chemical reaction. These wet (dry) conditions were also well represented by low (high) solar radiation and high (low) total cloud cover (Fig. 3g,h, respectively). The solar radiation continues to decrease before high concentration day (Day 0) and the cloud cover increases in humid condition.

### Responsible synoptic conditions for aqueous phase reaction

The characteristics of weather environments favorable for high concentrations were examined together with synoptic conditions. Geopotential height and RH were analyzed to investigate comprehensively the changes in dynamic and thermodynamic mechanisms during the cold season. Since high concentration was resolved after Day 0, this study focused on the features of two previous days to the high concentration episode.

**Figure 4:**
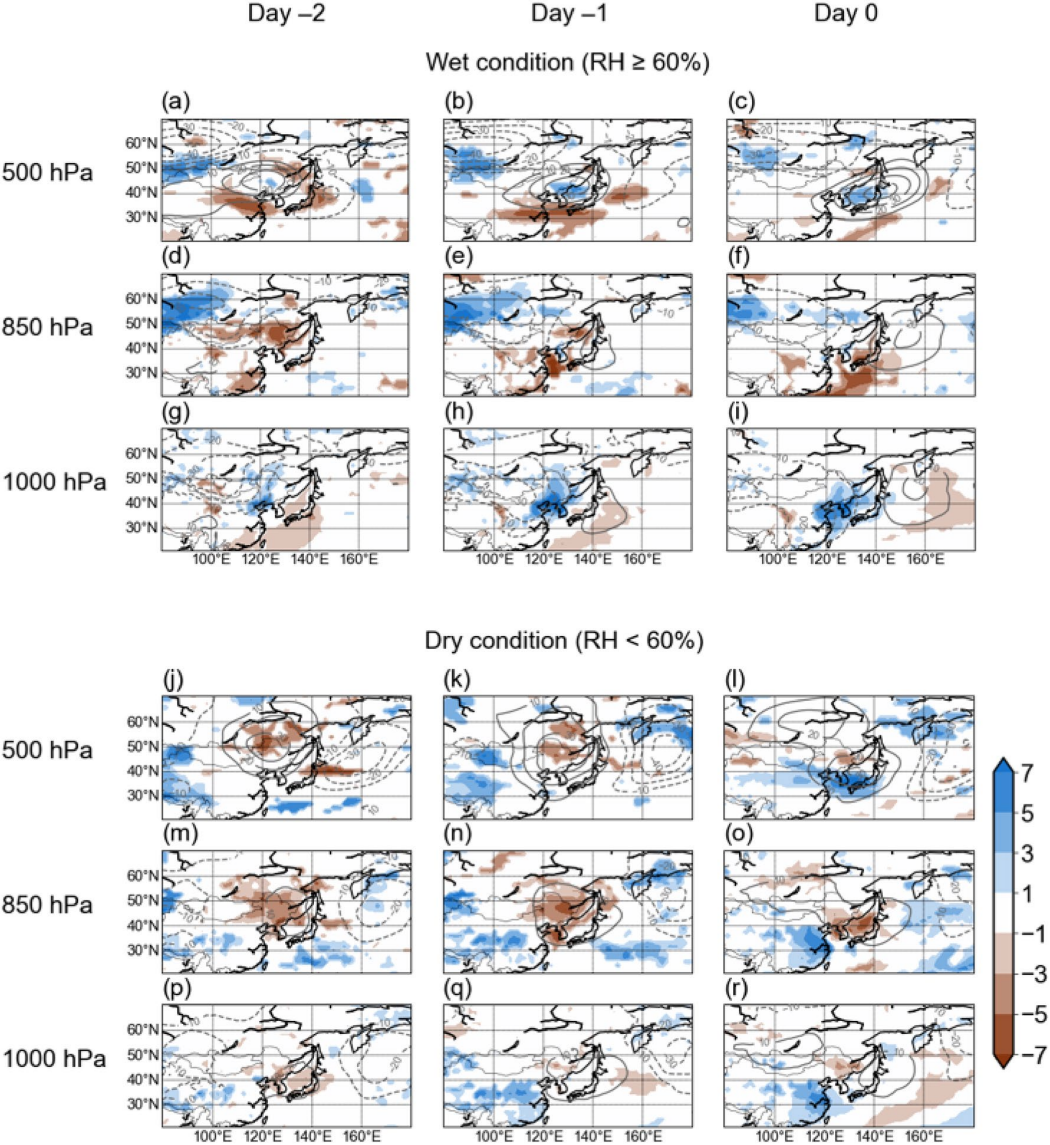
Composite of anomalous RH (shades) and geopotential height (contour) at 1000-, 850-, and 500-hPa for high-PM_2.5_ episodes under wet (upper panel) and dry (lower panel) condition in Day-2, Day-1, and Day 0 of high-PM_2.5_ episodes. The anomalies were calculated against the daily climatology to remove seasonality of meteorological variables. Only statistically significant anomalies in RH at the 90% confidence level are shown as color shades.

In both wet and dry cases, the anomalous high pressure at 500 hPa level was predominant over the Korean Peninsula from Day − 2 to Day 0 (Fig. 4a–c,j–l). However, the low- and high-pressure anomalies were observed in the lower troposphere (850 hPa and 1000 hPa) for humid (Fig. 4d–i) and dry cases (Fig. 4m–r), respectively. In the wet condition, a wide cyclonic circulation in the lower troposphere over the northeastern of China and Mongolia was observed on Day − 2. This low-level cyclonic circulation system moved to the east and eventually arrived in the Korean Peninsula on Day 0. The spatial distribution of geopotential height caused southerly on the Korean Peninsula and increased in 1000-hPa RH, resulting in an active environment for aqueous phase reaction. In contrast, anticyclonic circulation was located on the Korean Peninsula on Day 0 in the dry cases. The synoptic system was stagnant and produced dry conditions associated with barotropic high pressure over the Korean Peninsula^8,11–13,15–17^. Under these weather environments, solar radiation was increased by clear sky and gas phase reaction was active. Thus, the main reaction of secondary aerosol generation varied depending on the synoptic system.

## Discussion

This study investigated the developing mechanism of high PM_2.5_ concentration by secondary particle formation in humid condition through aqueous phase reaction in addition to photochemical reaction. It reveals that high concentration episodes could be occurred by oxidation even if the direct aerosol emission would be prohibited and atmospheric stagnation by blocking was not produced. The effect of temperature and RH on the secondary particle generation found in this study provided it possible to anticipate the future air quality of the Korean Peninsula corresponding with climate change and to consider countermeasures. Assuming that other factors such as emissions and/or atmospheric circulations are unchanged, it is expected that the aqueous phase reaction will be more active due to forthcoming warmer and wetter environments, which can contribute to worsening of air quality in the future. From the perspective of compound hazard according to the global warming, the impact of cold surge becomes more serious rather^56^ and it would be vulnerable to the high PM concentration episodes in the future^57^. This study suggests that the importance of thermodynamic variables such as temperature and humidity as well as the various precursors of aerosol should be recognized and used for prediction. Therefore, this study is expected to contribute to the improvement of understanding of compound hazards and the preparation of measures to reduce the social damages.

## Conclusion

Nowadays, severe air quality problem threatens human health is regarded as compound hazards causing ancillary damages and social costs. This study examined the environmental condition for high PM_2.5_ concentration focused on oxidation activity. Among the atmospheric chemical reactions for forming aerosol, the aqueous phase reaction of SIAs stimulated by water vapor in atmosphere showed greater contribution than other factors if the RH exceeded 60%. Although it is well known that secondary reaction could be active by photochemical reaction under the clear sky and stagnant synoptic system, the present results suggested the possibility of other contribution to aerosol formation even in a situation with little solar radiation. If the amount of water vapor in the atmosphere increases due to future warmer climate, the aqueous phase reaction is expected to be more active. This study warns that we may be exposed by high PM_2.5_ concentrations at any time due to increase both gaseous and aqueous phase reactions in the future.

## Data Availability

The air quality dataset used herein are available at https://www.airkorea.or.kr/. The meteorological datasets were obtained from https://data.kma.go.kr/cmmn/main.do. The reanalysis dataset was obtained from https://www.ecmwf.int/en/forecasts/datasets/reanalysis-datasets/era5.
